# CXCL12 May Drive Inflammatory Potential in the Ovine Corpus Luteum During Implantation

**DOI:** 10.1007/s43032-021-00791-0

**Published:** 2021-11-09

**Authors:** Stacia Z. McIntosh, Kelsey E. Quinn, Ryan L. Ashley

**Affiliations:** 1 Department of Animal and Range Sciences, New Mexico State University, MSC 3-I, PO Box 30003, Las Cruces, NM 88003 USA; 2 Department of Cell Biology and Physiology, University of North Carolina, Chapel Hill, NC USA

**Keywords:** Corpus luteum, Cytokines, Chemokines, Granulosa cells, Immunology, Molecular biology, Progesterone, Reproductive immunology, Ruminants, Sheep

## Abstract

Adequate corpus luteum (CL) function is paramount to successful pregnancy. Structural and functional CL integrity is controlled by diverse cell types that contribute and respond to the local cytokine milieu. The chemokine ligand 12 (CXCL12) and receptor, CXCR4, are modulators of inflammation and cell survival, but little is understood about CXCL12-CXCR4 axis and CL functional regulation. Corpora lutea from control nonpregnant ewes (*n* = 5; day 10 estrous cycle (D10C)) and pregnant ewes (*n* = 5/day) on days 20 (D20P) and 30 (D30P) post-breeding were analyzed for gene and protein expression of CXCL12, CXCR4, and select inflammatory cytokines. In separate cell culture studies, cytokine production was evaluated following CXCL12 treatment. Abundance of CXCL12 and CXCR4 increased (*P* < 0.05) in pregnant ewes compared to nonpregnant ewes, as determined by a combination of quantitative PCR, immunoblot, and immunofluorescence microscopy. CXCR4 was detected in steroidogenic and nonsteroidogenic cells in ovine CL, and select pro-inflammatory mediators were greater in CL from pregnant ewes. In vitro studies revealed greater abundance of tumor necrosis factor (TNF) following CXCL12 administration (*P* = 0.05), while P4 levels in cell media were unchanged. Fully functional CL of pregnant ewes is characterized by increased abundance of inflammatory cytokines which may function in a luteotropic manner. We report concurrent increases in CXCL12, CXCR4, and select inflammatory mediators in ovine CL as early pregnancy progresses. We propose CXCL12 stimulates production of select cytokines, rather than P4 in the CL to assist in CL establishment and survival.

## Background

Progesterone (P4) synthesis and secretion during early pregnancy in mammals is the responsibility of the corpus luteum (CL), a dynamic, transient endocrine gland that develops in place of the follicle following ovulation. Structural condition of the CL throughout the estrous cycle and early pregnancy is dependent on a closely regulated relationship between cells comprising the CL, which act on one another in both autocrine and paracrine fashion to regulate luteinization, steroidogenesis, and angiogenesis [1–3]. Luteal functional integrity and luteolysis during the estrous cycle depend on local production of inflammatory mediators by resident and recruited cells [4–7], yet gaps in knowledge exist regarding molecular expression and functions of cytokines and chemokines in the CL during early pregnancy.

The ligand-receptor pair C-X-C motif chemokine ligand 12 (CXCL12) and C-X-C motif chemokine receptor 4 (CXCR4) is implicated in both implantation and placentation as reported in sheep [8, 9], humans [10, 11], baboons [12], and mice [13], and we along with others have reported that activation of CXCR4 by CXCL12 regulates inflammatory cytokine abundance at the fetal-maternal interface and in other tissues [14, 15]. Potential roles for this chemokine duo have additionally emerged in the ovary; human granulosa cells express CXCR4, and CXCL12 is found in follicular fluid [16], which may improve granulosa cell viability. Both members of this chemokine-receptor pair have been characterized in equine, bovine, and ovine preovulatory follicles, and this axis plays a pivotal role in oocyte nuclear maturation in sheep [17, 18]. In humans, follicular CXCR4 abundance is stimulated by the ovulation-inducing luteinizing hormone surge and may aid in ovulation [19]. Indeed, chemokine signaling reportedly contributes to luteal lifespan [20] and may be a key regulator moderating the CL cytokine milieu. We demonstrated ovine CL express CXCR4 [21], but the CXCL12-CXCR4 signaling axis has not been explored in terms of luteal function during early gestation.

Suboptimal luteal P4 synthesis is a critical culprit in pregnancy loss [22], especially early in gestation when the CL is the sole source of P4. A majority of studies investigating the CL with respect to cytokines/chemokines have focused on the estrous cycle and/or luteolysis, which creates a major barrier for thoroughly appreciating luteal establishment and functionality during early pregnancy and its implications with respect to uterine receptivity and proper implantation and placentation early in gestation. A better comprehension of chemokine signaling in the CL during early pregnancy yields the potential to further the progress of therapeutic methods that will improve reproductive efficiency in both livestock and humans. Our current understanding that CXCL12-CXCR4 signaling during early pregnancy encourages trophoblast survival and modulates the fetal-maternal microenvironment [8, 23, 24] may simultaneously extend to luteal survival and function. In this study, we characterized the expression and abundance changes for CXCL12, CXCR4, and inflammatory cytokines in the ovine CL of early pregnancy and, further, investigated the influence of CXCL12-CXCR4 signaling on cytokine production in vitro.

## Methods

### Animal Procedures and Tissue Collection

Corpora lutea used for the current study were collected from our previously published research, and all procedures involving animals were approved by the New Mexico State University Institutional Animal Care and Use Committee (#2011–023) [9]. Briefly, synchronized, unbred ewes were anesthetized with 20 mg/kg body weight of sodium pentobarbital (48,126; Vortech Pharmacy, Dearborn, MI, USA) on day 10 of the estrous cycle (D10C, nonpregnant control; *n* = 5). Subsets of bred ewes were subjected to the same procedure on either day 20 (D20P; *n* = 5) or 30 (D30P; *n* = 5) post-breeding, and pregnancy was confirmed by presence of a conceptus. From each ewe, the reproductive tract was removed via mid-ventral laparotomy, and CL were collected with sterile technique, snap frozen in liquid nitrogen, and stored at − 80 °C for subsequent RNA and protein isolation. Cross-sections of CL (0.5 cm thick) were obtained using a sterile razor blade and immersed in 4% paraformaldehyde for 24 h, paraffin-embedded according to standard histological procedures, and mounted onto glass slides (AML Laboratories, Saint Augustine, FL, USA).

### Cell Culture

KGN cells, an established steroidogenic granulosa-like cell line [25] (a generous gift from Dr. Jennifer Hernandez Gifford; New Mexico State University, Las Cruces, NM), were grown in complete medium consisting of DMEM/F12 (Gibco by Life Technologies, Grand Island, NY, USA) supplemented with 10% fetal bovine serum (FBS) and 1% antibiotic blend (100 I.U. penicillin and 100 µg/mL streptomycin) and maintained in atmospheric air with 5% CO_2_ at 37 °C. Cells were seeded at 350,000 cells/well into 6-well tissue culture plates and allowed 24 h to settle and adhere to the wells before their media was replaced with medium lacking FBS. Approximately 24 h later, cells were treated with CXCL12 (300-28A; PeproTech, Rocky Hill, NJ, USA) at varying concentrations (0, 10, 20, 50, or 100 ng/mL). After 24 h in culture, cells were washed in phosphate-buffered saline (PBS), and protein was harvested using 300 µL of radioimmunoprecipitation assay (RIPA) buffer (50 mM Tris base, 2 mM EDTA, 150 mM NaCl, 0.1% SDS, 1.0% Triton X-100) supplemented with protease and phosphatase inhibitors (Roche Applied Science, Mannheim, Germany). Media was collected, centrifuged at 10,000 × *g* at 4 °C, and supernatants were isolated and stored at − 20 °C until P4 analysis. Cell lysates were centrifuged at 12,000 × *g* for 5 min at 4 °C, and supernatants were transferred into fresh tubes for storage at − 20 °C until protein quantification and immunoblot analysis.

### Progesterone RIA

P4 in cell culture media was measured by the New Mexico State University Endocrinology Laboratory (Las Cruces, NM, USA) using a commercially available radioimmunoassay (RIA) kit equipped with antibody-coated tube technology (Siemens Medical Solutions Diagnostics, Los Angeles, CA, USA). The intra-assay CV was 6.4%.

### RNA Isolation and Quantitative PCR (qPCR)

Total RNA was extracted from CL tissue using 1 mL of Tri Reagent (Molecular Research Center Inc, Cincinnati, OH, USA) per 100 mg of tissue according to the manufacturer’s instructions and eluted with nuclease-free water. Ribonucleic acid was treated with DNase using the TURBO DNA-free kit (Ambion, Foster City, CA, USA) to eliminate genomic DNA contamination. Specifications of RNA quantity and purity were determined using a NanoDrop-2000 spectrophotometer (Thermo Fisher Scientific, Waltham, MA, USA). Ribonucleic acid samples were stored at − 80 °C until further analysis.

Complementary DNA (cDNA) was synthesized using the iScript cDNA synthesis kit (Bio-Rad Laboratories, Hercules, CA, USA) with 1 μg of RNA for each sample according to the manufacturer’s instructions. Samples were diluted to a final volume of 100 μL with nuclease-free water. Analysis of qPCR was performed with a CFX96 Touch Real-Time PCR Detection System using iQ SYBR Green Supermix (Bio-Rad Laboratories) and primers listed in Table 1. Amplicon size for all targets was between 100 and 150 bp, and amplification efficiencies were validated for each primer using a tenfold dilution series of cDNA for each primer set. The qPCR protocol began with 95 °C for 3 min and then 39 cycles of 95 °C (30 s), 55 °C (30 s), and 72 °C (15 s) and completed with a melt curve. Glyceraldehyde phosphate dehydrogenase (*GAPDH*) amplicon did not change across days or pregnancy status and was used to normalize each target mRNA. Data are represented by graphing 2^−ΔΔCt^ values calculated for each target gene.

**Table 1:** Ovine primer sequences for each target gene

Target	Sequence	Accession no
*GAPDH*	5′-TGACCCCTTCATTGACCTTC-3′ 5′-CGTTCTCTGCCTTGACTGTG-3′	NM_001190390
*CXCL12*	5′-CCTTGCCGATTCTTTGAGAG-3′ 5′-GGTCAATGCACACTTGCCTA-3′	NM_001113174
*CXCR4*	5′-GGGATCCGTATATTCACTTCCGA-3′ 5′-ATTTTCCTCCCGGAAGCAGG-3′	NM_174301
*IL10*	5′-GGCGCTGTCATCGTTTTCTG-3′ 5′-ACACCCCTCTCTTGGAGCAT-3′	NM_001009327.1
*IL12A*	5′AGCCACGAATGAGAGTTGCC-3′ 5′-TCCAGAAGACAGACAATGCCC-3′	NM_001009736.1
*IFNG*	5′-GGCTGATTCAAATTCCGGTGG-3′ 5′-TCTCCGGCCTCGAAAGAGAT-3′	NM_001009803.1
*TNF*	5′-GTAGCCCACGTTGTAGCCAA-3′ 5′-TCAGGTAAAGCCCGTCAGTG-3′	NM_001024860.1
*TGFB1*	5′-AGAAGGCTTTCGCCTCAGTG-3′ 5′-CCGGAACTGAACCCGTTGAT-3′	NM_001009400.1

### Protein Isolation and Immunoblotting

Corpora lutea were homogenized using 1 mL of RIPA buffer supplemented with phosphatase and protease inhibitor per 100 mg of tissue. Isolates were placed on ice for 15 min, centrifuged at 12,000 × *g* for 10 min at 4 °C, and protein concentrations within the supernatant were determined by bicinchoninic acid (BCA) protein assay. Lysates were stored at − 80 °C until immunoblot analysis.

Equal amounts of CL protein were separated by SDS-PAGE using 10% polyacrylamide gel under either reducing or non-reducing conditions and transferred to methanol-activated polyvinyl difluoride membranes. Membranes were blocked in 5% non-fat milk or 5% bovine serum albumin (BSA) made in Tris-buffered saline with Tween-20 (TBST; 68.4 mM Tris base, 10 mM NaCl, 0.10% Tween-20, pH 7.6) for 1 h at room temperature and subsequently incubated overnight at 4 °C with primary antibody. All antibodies used for immunoblot analyses are described in Table 2. Primary antibodies were diluted in either 5% non-fat milk or 5% BSA made in TBST with dilutions ranging from 1:1000 to 1:2000. The following day, membranes were washed twice for 10 min in TBST and subsequently incubated with appropriate IgG-horseradish peroxidase-conjugated secondary antibody for 1 h at room temperature. After washing each membrane for 2 × 10 min, the Immun-Star™ WesternC™ kit (Bio-Rad Laboratories) was used to visualize proteins of interest on the ChemiDoc™ XRS with Image Lab Software (Version 3, Bio-Rad Laboratories), and background was subtracted from all bands of interest. Equal loading was confirmed for each immunoblot using GAPDH under the incubation conditions described above.

**Table 2:** Antibodies used to detect each protein of interest

Target	Company	RRID
GAPDH	3683; Cell Signaling Technology	AB_1642205
CXCL12	3530; Cell Signaling Technology	AB_2088167
CXCR4	sc-9046; Santa Cruz Biotechnology	AB_2245742
CXCR4^1^	ab124824; Abcam	AB_10975635
IL12	MCA1782EL; Bio-Rad Laboratories	AB_616909
IFNG	MCA1964; Bio-Rad Laboratories	AB_2123455
TNF	251,900; Abbiotec, San Diego, CA	AB_10636377
Mouse IgG	sc-2005; Santa Cruz Biotechnology	AB_631736
Rabbit IgG	sc-2004; Santa Cruz Biotechnology	AB_631746
Rabbit IgG^1^	A1108; Life Technologies	AB_143165
ZAP70 pY319	65E4; Cell Signaling Technology	AB_2218658

^1^Antibodies used for immunofluorescence.

### Immunofluorescence Staining and Microscopy

Immunostaining of CXCR4 on 5-µm sections of paraffin-embedded CL tissue was performed using the Sequenza slide rack and cover plate system (Thermo Fisher Scientific). All dilutions were made in Tris-buffered saline (TBS; 68.4 mM Tris base, 10 mM NaCl, pH 7.4), and treatments were applied using the Sequenza system at room temperature, following assembly, unless otherwise noted. Slides were deparaffinized through submersion in a series of dilutions: Histo-Clear (2 × 5 min), 1:1 Histo-Clear and ethanol (3 min), 100% ethanol (2 × 3 min), 95% ethanol (3 min), 70% ethanol (3 min), 50% ethanol (3 min), and distilled water (5 min). Antigen retrieval was performed on tissue slides by boiling samples in sodium citrate (10-mM sodium citrate buffer, 0.05% Tween-20, pH 6) for 7 min. After submerging in cold running water for 10 min, slides were assembled in the Sequenza, washed twice using 0.025% Triton X-100 (TBS-Tr), and blocked for 1 h in TBS supplemented with 10% normal goat serum and 1% BSA followed by a 30-min incubation with Image-IT FX Signal Enhancer (136,933; Thermo Fisher Scientific) and subsequent 1-h incubation at 37 °C with CXCR4-specific antibody (1:400) in 1% BSA. Sections were then washed twice with TBS-Tr and incubated for 1 h with an Alexafluor 488-conjugated secondary antibody (1:200) diluted in 1% BSA. After rinsing each section twice with TBS, Fluoromount (F4680; Millipore Sigma, St. Louis, MO, USA) supplemented with 4,6-diaminidino-2-phenylindole (DAPI; Life Technologies) was added to each section, and slides were fitted with a coverslip. At least 5 areas of at least 2 sections per animal were visualized using an Axio Observer.Z1 (Carl Zeiss Microscopy, Oberkochen, Germany).

### Statistical Analysis

All statistical analyses were performed using GraphPad Prism (Version 8; GraphPad Software, Inc., La Jolla, CA, USA). For gene expression, the cycle threshold (Ct) value for each gene of interest was normalized to that of *GAPDH* for each sample using the ΔCt method [26], and 2^−ΔΔCt^ data were subjected to statistical analysis. Chemiluminescent signals for immunoblots were quantified using Image Lab Software (version 4.1; Bio-Rad Laboratories) to obtain optical densitometry values for each band of interest; immunoblot data were normalized by dividing the mean intensity of the band corresponding to the target protein by that of the loading control. All data were analyzed using one-way analysis of variance (ANOVA), followed by either Dunnett’s or Tukey’s multiple comparison procedure, as appropriate, when significance was observed. Changes were considered significant when *P* ≤ 0.05 and tendency were accepted when *P* < 0.1.

## Results

### Abundance of Inflammatory Mediators in Ovine CL Across Early Gestation

**Fig. 1:**
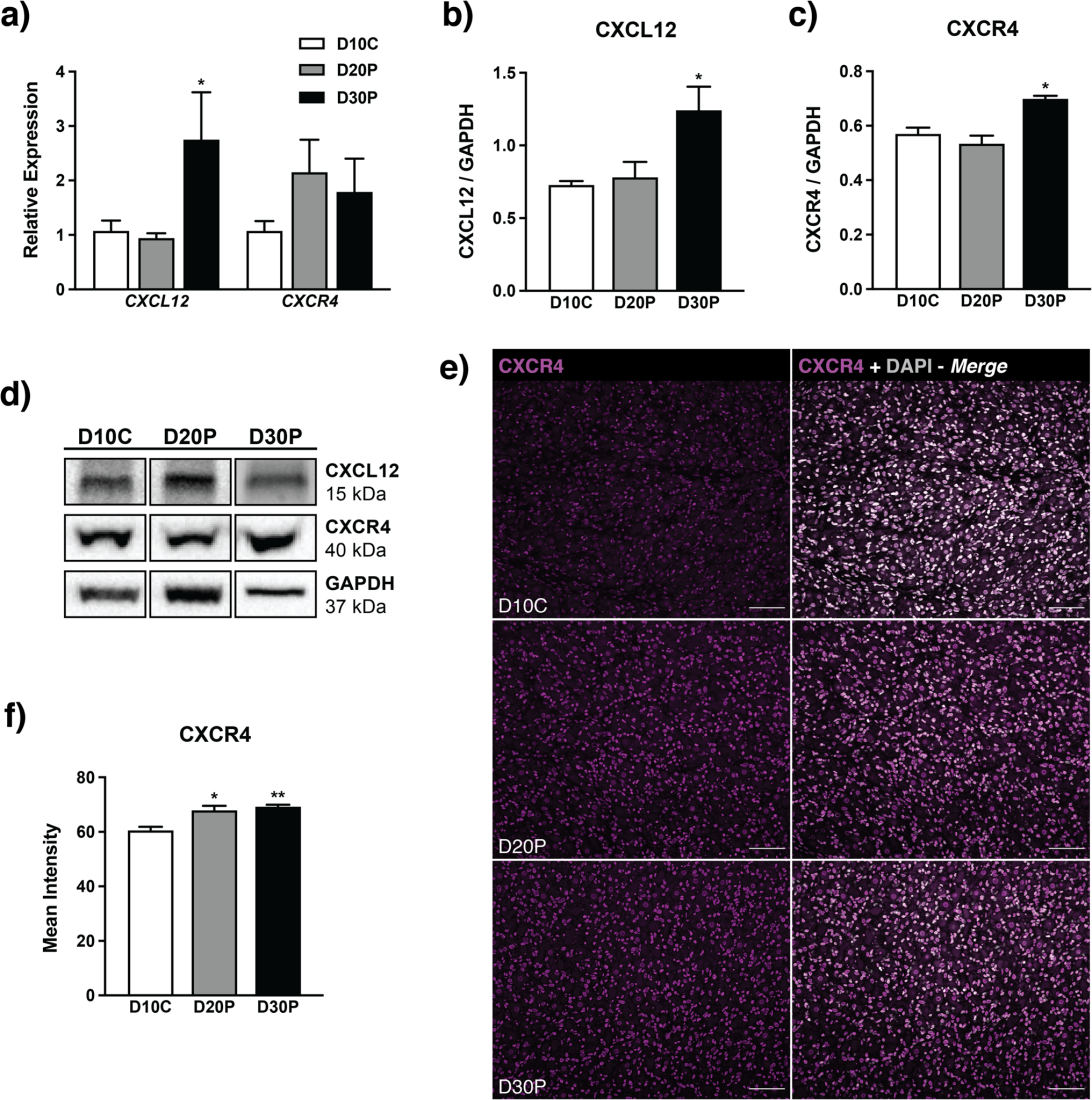
CXCL12 and CXCR4 in ovine corpora lutea on D10C, D20P, and D30P. **a** Gene expression for *CXCL12* and *CXCR4*. Protein abundance for **b** CXCL12 and **c** CXCR4 after normalizing to GAPDH optical densitometry values, followed by **d** corresponding representative immunoblots. **e** Representative micrographs of luteal cross-sections from all days sampled, displaying immunoreactive CXCR4 (magenta) and 4′,6-diamidino-2-phenylindole (DAPI; gray) as nuclear stain. Bars symbolize 75 µm. **f** Mean gray value quantitation of immunoreactive CXCR4 in tissue sections. Data represent the mean ± SEM, and asterisks indicate significance at *P* < 0.05 (*) or *P* < 0.01 (**)

**Fig. 2:**
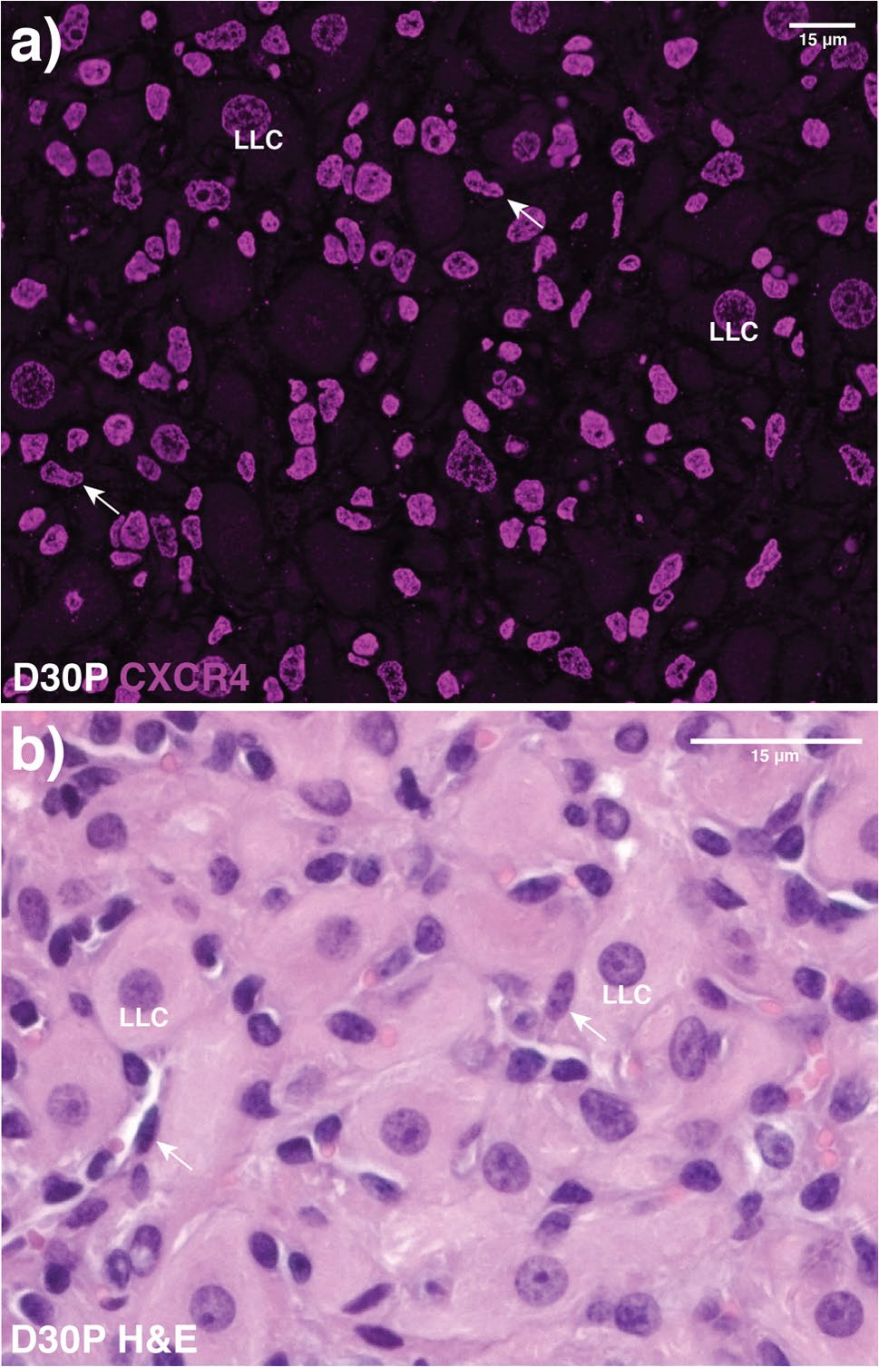
CXCR4 immunoreactivity and cellular composition of ovine corpora lutea at D30P. **a** Representative micrograph of CXCR4 (magenta) immunostaining among several luteal cell types, including large luteal cells (LLC) and fibroblasts (arrows). **b** Representative brightfield image of H&E-stained sections showing general morphology of the luteal microenvironment. Bars: 15 µm

To elucidate possible influence of CXCL12-CXCR4 signaling on luteal maintenance during early pregnancy, it was important to first characterize presence of both members of the signaling pair. To this end, Fig. 1a reveals greater mRNA expression of *CXCL12* on D30P compared to D10C (*P* < 0.05), while *CXCR4* transcript remained similar across all days analyzed. Protein abundance was detected by immunoblot, with elevated levels of both CXCL12 and CXCR4 on D30P (*P* < 0.05) compared to nonpregnant ewes on D10C (Fig. 1b and c). Immunoreactive CXCR4 was additionally detected using immunofluorescence and was greater on D20P (*P* < 0.05) and D30P (*P* < 0.01) compared to D10C (Fig. 2e and f). Moreover, we observed perinuclear CXCR4 staining detected in both steroidogenic (large luteal cells) and nonsteroidogenic cell types in ovine CL (Fig. 2).

**Fig. 3:**
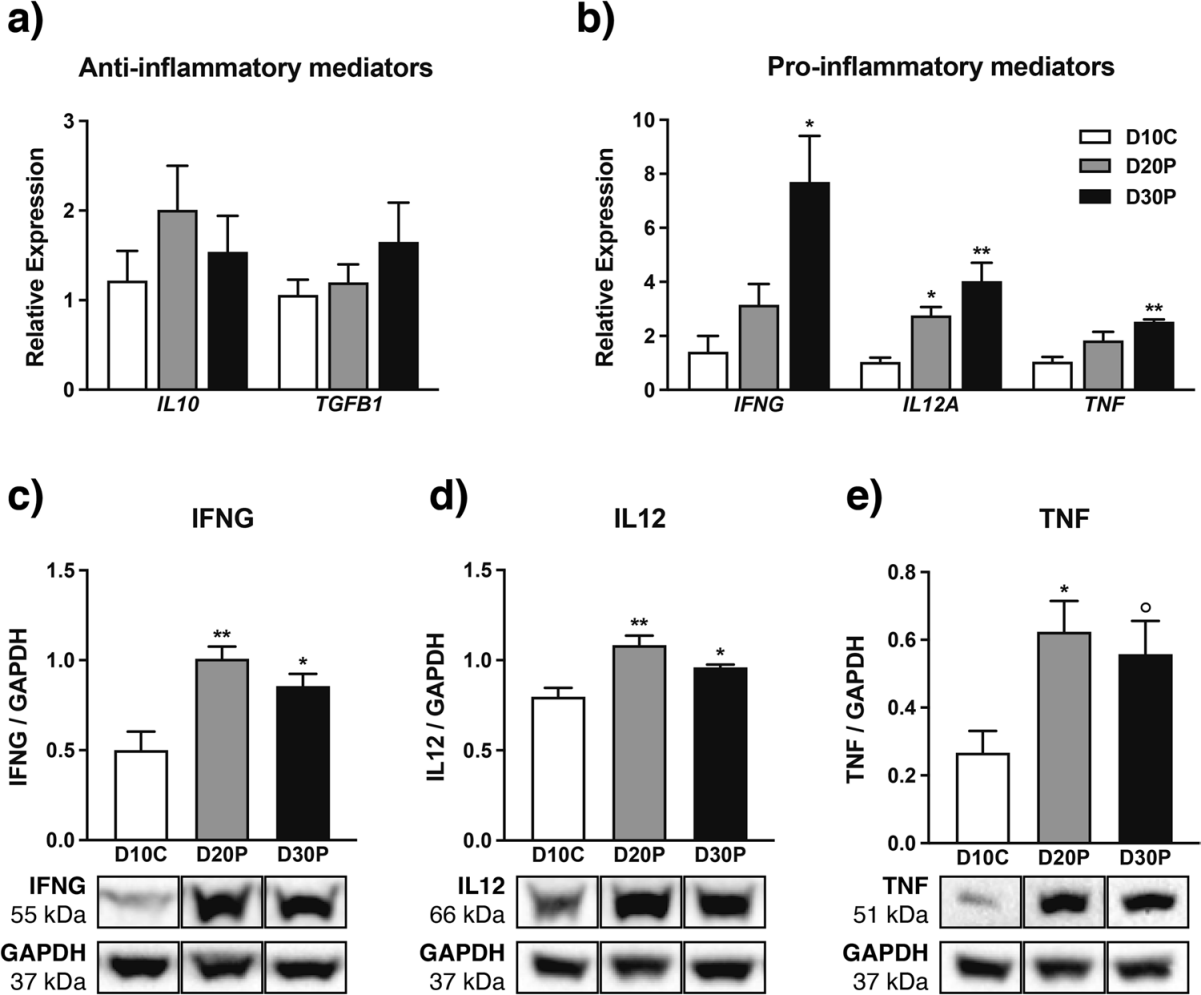
Inflammatory potential of ovine corpora lutea on D10C, D20P, and D30P. Transcript expression of **a** anti-inflammatory and **b** pro-inflammatory cytokines. Protein levels for **c** IFNG, **d** IL12, and **e** TNF, with each graph followed by representative immunoblot for both protein of interest and GAPDH (loading control) used for normalization. Data represent the mean ± SEM, and asterisks indicate significance at *P* < 0.05 (*) or *P* < 0.01 (**) and tendency when *P* < 0.1 (°) compared to D10C

We analyzed gene and protein expression of select inflammatory cytokines using qPCR and immunoblotting, respectively. Transcript for *IL10* and *TGFB1* was present in CL from both pregnant and cycling ewes but did not differ with pregnancy status (Fig. 3a). Contrary, pro-inflammatory *IFNG* expression in CL rose on D30P (*P* < 0.05), *IL12A* on D20P (*P* < 0.05) and D30P (*P* < 0.01), and *TNF* on D30P (*P* < 0.01) compared to that of nonpregnant cycling ewes (Fig. 3b). Protein abundance for pro-inflammatory cytokines was confirmed using immunoblot, revealing greater levels of both IFNG and IL12 on D20P (*P* < 0.01) and D30P (*P* < 0.05) compared to cycling ewes (Fig. 3c and d). In a similar fashion, TNF abundance was greater on D20P (*P* < 0.05) and had a tendency for elevated levels on D30P compared to CL from nonpregnant ewes (*P* < 0.1) (Fig. 3e).

### ZAP70 pY319 Levels Decrease in Ovine CL as Pregnancy Progresses

**Fig. 4:**
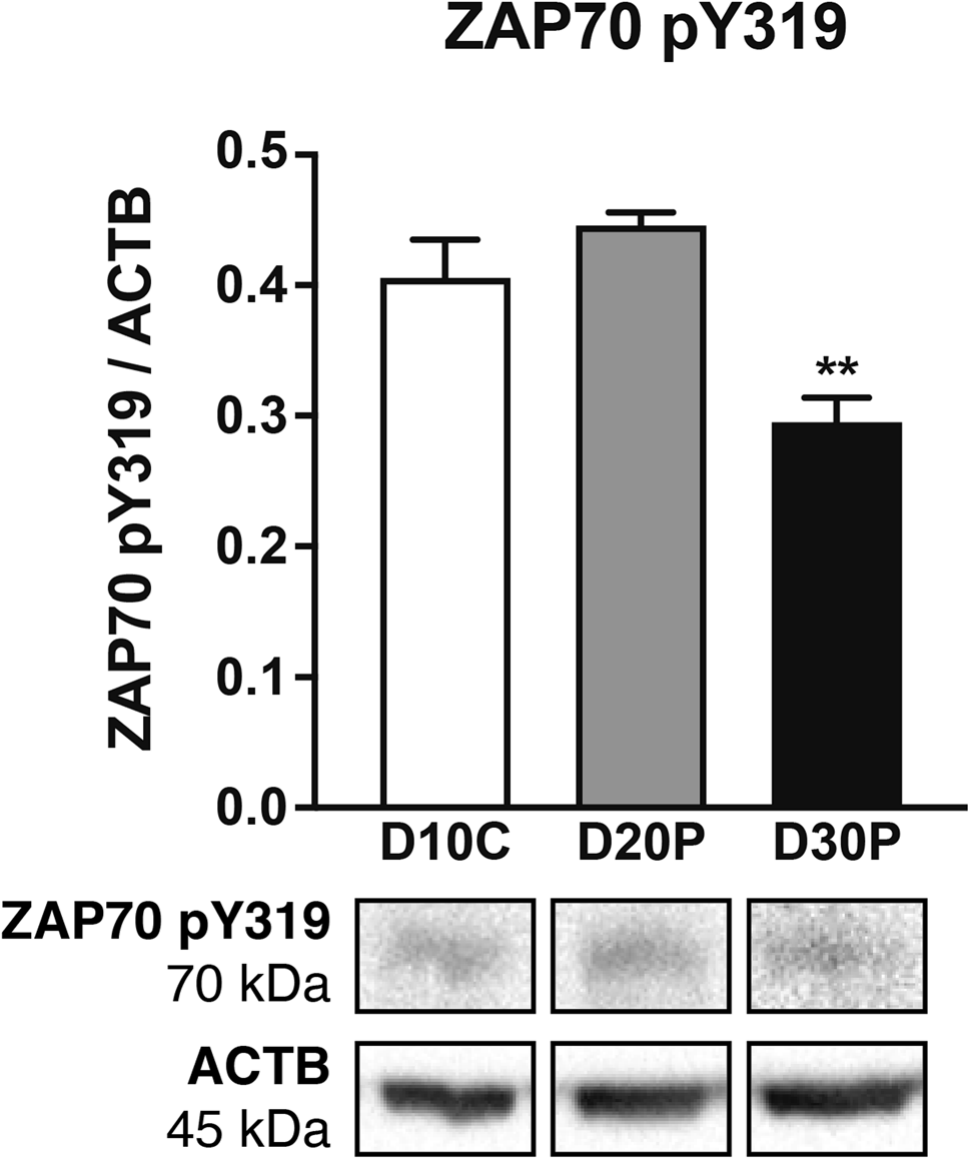
Abundance of phosphorylated ZAP70 in ovine corpora lutea across early gestation. Protein levels for ZAP70 pY319 on D10C, D20P, and D30P. Data represent the mean ± SEM, and each graph is followed by a representative immunoblot for both protein of interest and ACTB (loading control) used for normalization. Asterisks indicate significance at *P* < 0.01 (**) compared to D10C

Zeta chain of T cell receptor-associated protein kinase 70 (ZAP70) is a known mediator of CXCL12-CXCR4 signal transduction in lymphocytes [27, 28]. As such, we elected to examine phosphorylated ZAP70 (pY319) in CL from cycling and pregnant ewes to help discern the cell type(s) in which CXCL12-CXCR4 is predominantly signaling in the CL, whether it be immune cells or other nonhematopoietic cells present. Opposite to luteal CXCL12 and CXCR4 abundance, ZAP70 pY319 levels were down-regulated on D30P (*P* < 0.01) compared to CL of cycling ewes (Fig. 4).

### CXCL12 Stimulates TNF Protein Abundance in KGN Cells

**Fig. 5:**
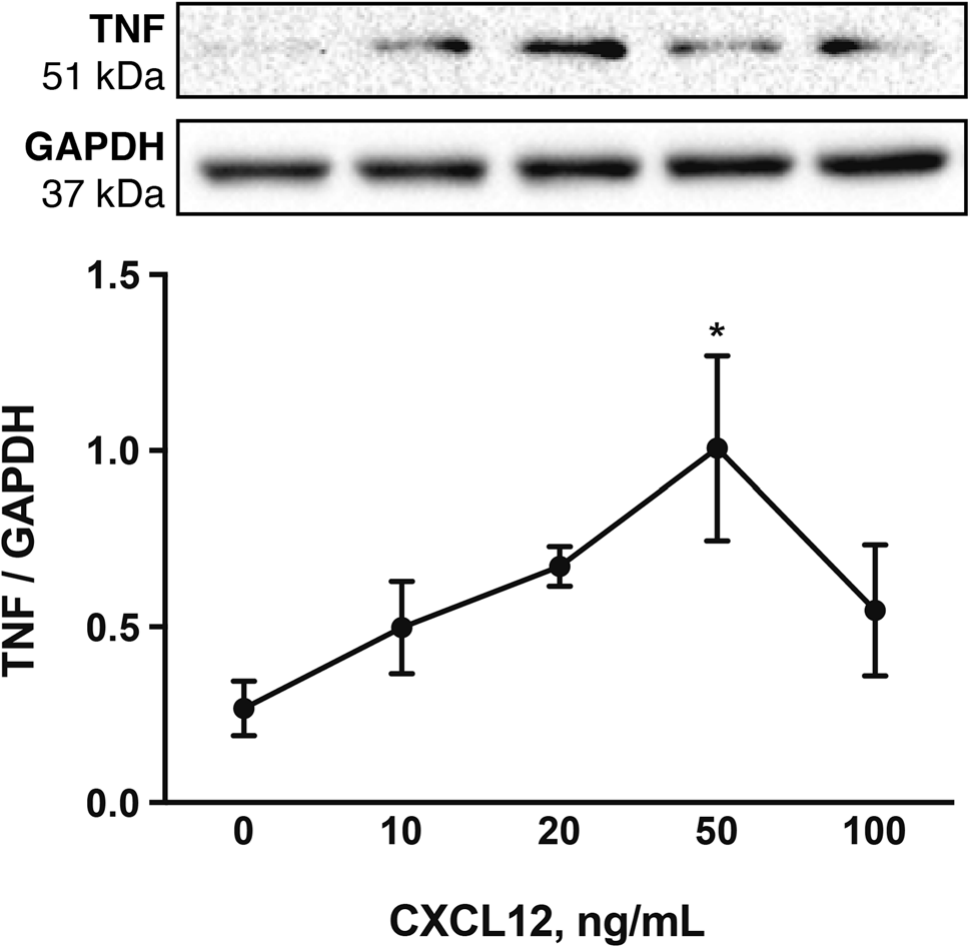
KGN TNF protein levels in response to increasing doses of CXCL12. Abundance of TNF following administration of CXCL12 at concentrations ranging from 0 to 100 ng/mL. Representative immunoblot for both protein of interest and GAPDH (loading control). Normalized optical densitometry values of TNF are graphed using the mean ± SEM. Asterisks indicate difference relative to control (0 ng/mL) at *P* < 0.05 (*). Experiments were repeated a minimum of 4 times

To determine if CXCL12 directly alters cytokine synthesis in the CL, in vitro studies were completed using the steroidogenic KGN cell line [25]. After confirming CXCR4 presence, we evaluated TNF abundance following exposure to increasing doses of CXCL12. After 24 h in culture, TNF abundance was similar to that of baseline levels (0 ng/mL CXCL12 treatment) for all doses of CXCL12, aside from an increase in response to 50 ng/mL CXCL12 (*P* < 0.05) (Fig. 5).

## Discussion

As sole contributor of P4 throughout early gestation, presence and proper development of the CL are essential for establishing and maintaining an intrauterine environment appropriate for conceptus implantation and survival. Activation of the CXCL12-CXCR4 signaling axis is pivotal in regulating immune cell trafficking, vascularization, and inflammatory potential at the fetal-maternal interface, and similar control may also occur in the CL. Despite the known existence of this chemokine-receptor pair in the CL of pregnant sheep [21], its activity in the CL of pregnant animals is poorly understood.

Reports of CXCL12-CXCR4 activity in the ovary thus far have focused on chemotactic properties related to immune cell recruitment and the resulting implications regarding folliculogenesis [16, 19, 29, 30]. However, an increase in follicular fluid CXCL12 levels correlates with luteal formation as reported by Nishigaki and colleagues [31], who hypothesized that CXCL12 may facilitate not only follicular development, but luteinization as well. Our characterization of elevated levels of luteal CXCL12 and CXCR4 in pregnant but not nonpregnant ewes in the present study reinforces this notion. CXCR4 cellular localization was chiefly along the nuclear membrane, an indicator of activation linked to signaling events inducing migration and invasiveness both in vitro and in vivo [32, 33]. More recently, a nuclear localization sequence has been identified within CXCR4 that permits its transport to the nucleus, where the CXCR4 remains functional and responsive to CXCL12 [34]. Though the mechanism(s) at play in our present account remains a question, these findings imply a participating role of CXCL12-CXCR4 signaling in luteal integrity during early pregnancy, whether it be structural, functional, or a combination of the two.

A number of cytokines including IFNG, IL10, IL12, and TNF have been identified in follicular fluid and participate in granulosa cell proliferation, oocyte viability, and ovulation [35] and therefore are likely targets of luteal CXCL12-CXCR4 activity. Transcript was detected and remained similar for both *IL10* and TGFB1 across all days analyzed. A principal function of IL10 is that of mediating abundance of pro-inflammatory molecules, IL10 may remain present at low levels to prevent excessive inflammation caused by IL12, IFNG, and possibly TNF [36]. Unchanged *TGFB1* expression is consistent with a recent characterization of the bovine luteal microenvironment by Gadsby and colleagues [37]. In murine models, TGFB1-deficient females exhibit substantially reduced fertility, the consequence of a combination of defective embryonic development and impaired ovarian function [38, 39]. Indeed, *Tgfb1*^*−*^*/*^*−*^ mice have fewer CL that display diminished functionality; serum P4 levels among these animals is less than their *Tgfb1*^+^*/*^+^ counterparts, even after accounting for the difference in CL number [39]. Despite this, reports using domestic animal models refer to TGFB1 as luteolytic—PGF2A administration, both in vivo and in vitro, increases *TGFB1* transcript [40] in cattle, and ovine luteal regression is characterized by 19 differentially expressed genes from the TGFB1 pathway [41]. In primary cells isolated from bovine CL, TGFB1 treatment suppresses P4 secretion by luteal cells [40] and induces microvascular disassembly in luteal endothelial cells [42]. While this appears in conflict with descriptions implicating TGFB1 as a driver of placental angiogenesis [43], it also serves as a reminder of the dynamic nature of inflammatory mediators and their effects on different reproductive tissues.

Pro-inflammatory IFNG is crucial to vascular remodeling at the murine implantation site [44], and trophoblast-derived IFNG modifies tight junctions in maternal endometrium between days 12 and 20 of porcine pregnancy [45]. A regulator of cell-mediated immune responses, IL12 mainly functions in recruitment and activation of cells with an inflammatory phenotype and induce expression of IFNG [36, 46–48]. The synergistic relationship between IL12 and IFNG is hypothesized to take place in the bovine CL [37], and our findings suggest a similar likelihood in an ovine model. As it stands, evidence of IL12 activity in the follicle is inconsistent; some fail to detect IL12 in follicular fluid [49, 50], and others positively correlate follicular IL12 with oocyte and embryo quality [51]. Still, there are several reports negatively associating IL12 follicular abundance to oocyte maturity, instead hypothesizing this molecule to be an indicator of poor IVF outcomes [52–55]. Much less is understood about the role of inflammatory mediators in the luteinization process compared to that of follicular development, which, itself, is limiting. However, along with IFNG, IL10, TNF, and TGFB1, IL12 is expressed during both formation and regression of the canine CL [56, 57]. While speculative, these findings, paired with concurrent elevated levels of CXCL12, CXCR4, and IFNG in the present study, suggest mediation of luteal IFNG by CXCL12-CXCR4 signaling either directly or indirectly, through elevated luteal IL12 abundance. Future studies will explore possible roles of IFNG in shaping the existing vasculature as well as its effect on presence and localization of tight junctions in the CL.

Similar to CXCL12, follicular expression of TNF is elevated as ovulation approaches [58], implying roles pertaining to both ovulation and the subsequent introductory stages of luteinization [59]. In the context of luteal angiogenesis, TNF promotes endothelial cell proliferation and angiogenic factor production in primary cells isolated from the early bovine luteal phase [60]. Likewise, in vivo TNF administration elevates secretion of vasodilators nitric oxide and prostaglandin E2 [61]. Regarding luteal function, TNF increases P4 production in cultured rat preovulatory follicles when administered at concentrations ranging from 1 to 100 ng/mL [62] and induces granulosa-lutein cell proliferation and P4 accretion in culture media [63]. Adding complexity to its actions, TNF exhibits dose-dependent effects: in vivo infusion of 10 µg of TNF in a bovine model not only boosts plasma P4, but also prevents spontaneous luteolysis and extends luteal function by over 9 days in comparison to infusion with higher and lower doses (1, 25, and 50 µg) of TNF and saline control [61]. TNF is, therefore, critical to CL angiogenesis, establishment, function, and lifespan; however, to expand our knowledge of TNF in the CL, we need to investigate the regulators driving local TNF production. Our present findings of increased TNF, CXCL12, and CXCR4 in the CL as pregnancy advances combined with our in vitro data of CXCL12-CXCR4 directly activating TNF production in steroidogenic cells further supports the CXCL12-CXCR4 axis as a regulator of TNF.

Further supporting the likelihood of pro-inflammatory cytokines’ positive impact on luteal function, immunoreactive TNF in the human CL is detected at the greatest levels in granulosa-lutein cells during the mid-luteal phase and decreases as luteal regression advances [64]. Nuclear localization of immunoreactive CXCR4 among large luteal cells in our present findings, along with concurrent elevated abundance of inflammatory cytokines during this physiological timeframe, may be regulated by CXCL12 signaling. Our in vitro findings support this hypothesis, at least in part, as exposure to physiological concentrations of CXCL12 boosted TNF synthesis in vitro. IFNG and IL12 were both detectable in cell lysate; however, their abundance was unchanged by increasing doses of CXCL12 ranging from 10 to 100 ng/mL (data not shown). A paucity currently exists in terms of presence and localization of IFNG and IL12 in the CL; it may be that CXCL12 is instead targeting luteal endothelial cells to drive production of IFNG. While these data posit CXCL12 as a driver of the demonstrated rise in luteal TNF levels, the mechanism, and source, of the concurrent increase in both IFNG and IL12 deserves additional exploration.

In T lymphocytes, CXCL12 induces association of CXCR4 and the T cell receptor to create a constitutively phosphorylated intracellular complex via the tyrosine kinase ZAP70, enabling prolonged extracellular signal-regulated kinase (ERK) activation as well as T cell migration [27, 65]. ZAP70 is a key enzyme that functions in lymphocyte activation and chemotaxis [28, 65]; once activated, ZAP70 autophosphorylates, creating docking sites for additional proteins that induce cytokine production [66–68]. For this reason, we focused on ZAP70 to evaluate whether the altered luteal inflammatory cytokine levels, which mirrored expression of CXCL12-CXCR4, were due to CXCL12-CXCR4 signaling in luteal lymphocytes. In contrast to the changes observed among inflammatory mediators in the CL with advancing pregnancy, less ZAP70 pY319 was detected in CL of ewes on D30P compared to D10C, suggesting CXCL12 was not directly acting on immune cells. The increase in cytokines, as well as CXCL12 and CXCR4, in CL may instead reflect altered synthesis by other cells such as steroidogenic luteal cells. Further, reduced ZAP70 pY319 could result from elevated P4 at this time during pregnancy, as P4 is known to decrease amounts of phosphorylated ZAP70 [68], or the simultaneous heightened production of inflammatory cytokines in the CL microenvironment. While IL12 treatment does not have an effect on ZAP70 phosphorylation [69], extended TNF treatment of T cells in culture down-regulates phosphorylated ZAP70 [70]. Whether the noted increase in TNF yielded less ZAP70 phosphorylation requires further investigation. Nevertheless, the decline in ZAP70 pY319 with gestation progression suggests CXCL12 may have direct actions on luteal cells. We propose that CXCL12 stimulates production of select cytokines, rather than P4 synthesis, by luteal cells as treatment of steroidogenic cells with CXCL12 increased TNF but P4 secretion remained unchanged compared to control (data not shown).

## Conclusions

In summary, our present findings serve as the first report of concurrent increases in CXCL12, CXCR4, and inflammatory mediators in the fully functional ovine CL as early pregnancy progresses. Although speculative, the distinct rise in TNF and IFNG suggests a luteotropic effect of these molecules, which may be under CXCL12-CXCR4 regulation. Luteal cells may be the key players in this story, as CXCL12 drives TNF production in progesterone producing cells and CXCR4 is present in luteal cells. The results described herein provide insight to the dynamic nature of the CL, whose structural and functional integrity is of vital importance to the establishment and maintenance of a healthy pregnancy. Still, further studies are required to verify the mechanism by which pro-inflammatory molecules engage in this series of events.

## Data Availability

The datasets used and/or analyzed during the current study are available from the corresponding author upon reasonable request.

## Abbreviations

BCA: Bicinchoninic acid assay
BSA: Bovine serum albumin
cDNA: Complementary deoxyribonucleic acid
CL: Corpus luteum
Ct: Cycle threshold
CXCL12: C-X-C chemokine ligand 12
CXCR4: C-X-C chemokine receptor 4
D10C: Nonpregnant control
D20P: Day 20 of pregnancy
D30P: Day 30 of pregnancy
ERK: Extracellular signal-regulated kinase
FBS: Fetal bovine serum
GAPDH: Glyceraldehyde phosphate dehydrogenase
IFNG: Interferon gamma
IL: Interleukin
PBS: Phosphate-buffered saline
NFKB: Nuclear factor kappa B
P4: Progesterone
RIPA: Radioimmunoprecipitation assay
TBS: Tris-buffered saline
TGFB: Transforming growth factor beta
TNF: Tumor necrosis factor
TNFR: Tumor necrosis factor receptor
TRAF: Tumor necrosis factor receptor-associated factor
VEH: Vehicle control
ZAP70: Zeta chain of T cell receptor-associated protein kinase 70
ZAP70 pY319: Phosphorylated ZAP70
